# Understanding Differences in Treatment Burden Among Older Adults with Multiple Chronic Conditions

**DOI:** 10.1093/geroni/igaf122.2279

**Published:** 2025-12-31

**Authors:** Anne Wang, Jie Liang, Lea Kiefer, Marcia Mecca, Lilian Dindo, Terri Fried, Aanand Naik

**Affiliations:** Michael E. DeBakey VA Medical Center, Houston, Texas, United States; UTHealth Houston, Houston, Texas, United States; Michael E. DeBakey VA Medical Center, Houston, Texas, United States; Yale School of Medicine, New Haven, Connecticut, United States; Michael E. DeBakey VA Medical Center, Houston, Texas, United States; West Haven VA Medical Center, West Haven, Connecticut, United States; UTHealth Houston, Houston, Texas, United States

## Abstract

Older adults managing multiple chronic conditions (MCCs) often need to balance various medications, treatments, and interventions, so this group of older adults is expected to experience healthcare burden. In this study, we examined the treatment burden of a group of 397 veterans who were older than 65 with three or more chronic conditions and/or ten or more medications. Of this group, 115/397 surprisingly reported no burden (NB) on the Treatment Burden Questionnaire (overall score = zero) while 282/397 reported an average score of 50 during baseline data collection. A chi-square test was run on different demographic variables to determine differences between participants reporting NB versus some burden (SB). NB and SB groups had older adults of similar race, gender, ethnicity, housing status, and educational level. However, older adults reporting NB more frequently reported perfect scores on the PROMIS questionnaire (NB: 48.7%; SB: 17.4%) and a 27/30 (90%) or better on the WBS questionnaire (NB: 52.2%; SB: 19.9%). Older adults reporting SB more frequently reported using assistive devices (NB: 20%; SB: 34.8%) and feeling as if every task was effortful (NB: 23.5%; SB: 40.8). These findings suggest that despite similar background and management of MCCs, older adults with minimal functional and mobility limitations often report NB. Furthermore, perceptions of treatment burden are related to perceptions of wellbeing and quality of life.

